# The Impact of Clinical Social Franchising on Health Services in Low- and Middle-Income Countries: A Systematic Review

**DOI:** 10.1371/journal.pone.0060669

**Published:** 2013-04-23

**Authors:** Naomi Beyeler, Anna York De La Cruz, Dominic Montagu

**Affiliations:** Global Health Group, University of California San Francisco, San Francisco, California, United States of America; The University of Auckland, New Zealand

## Abstract

**Background:**

The private sector plays a large role in health services delivery in low- and middle-income countries; yet significant gaps remain in the quality and accessibility of private sector services. Clinical social franchising, which applies the commercial franchising model to achieve social goals and improve health care, is increasingly used in developing countries to respond to these limitations. Despite the growth of this approach, limited evidence documents the effect of social franchising on improving health care quality and access.

**Objectives and Methods:**

We examined peer-reviewed and grey literature to evaluate the effect of social franchising on health care quality, equity, cost-effectiveness, and health outcomes. We included all studies of clinical social franchise programs located in low- and middle-income countries. We assessed study bias using the WHO-Johns Hopkins Rigour Scale and used narrative synthesis to evaluate the findings.

**Results:**

Of 113 identified articles, 23 were included in this review; these evaluated a small sample of franchises globally and focused on reproductive health franchises. Results varied widely across outcomes and programs. Social franchising was positively associated with increased client volume and client satisfaction. The findings on health care utilization and health impact were mixed; some studies find that franchises significantly outperform other models of health care, while others show franchises are equivalent to or worse than other private or public clinics. In two areas, cost-effectiveness and equity, social franchises were generally found to have poorer outcomes.

**Conclusions:**

Our review indicates that social franchising may strengthen some elements of private sector health care. However, gaps in the evidence remain. Additional research should include: further documentation of the effect of social franchising, evaluating the equity and cost-effectiveness of this intervention, and assessing the role of franchising within the context of the greater healthcare delivery system.

## Introduction

### Background

In many low- and middle-income countries (LMIC) the private sector is a primary source of health care [1], including for poor and rural populations [2], [3]. While the scale of the private sector continues to grow, challenges remain in the quality and distribution of private health services. Both the public and private sectors provide overall low quality care [2] and studies document very low quality in private sector services for malaria, tuberculosis, reproductive health, and children's health [4]–[6].

A number of interventions have been introduced to improve the quality of private health care [5].Social franchising is the fastest growing market-based health care intervention[7]; in 2011 53 franchise programs served 30 million patients globally, primarily in Asia and Africa [8]. Social franchising applies the principles of commercial franchising to provide widely distributed health services. Social franchises are networks of private providers, operating under contracts with a common agency and providing standardized products and services under a common brand. Social franchises typically include the following characteristics: outlets are operator-owned; outlets provide clinical services with or without franchise-branded commodities; and payments to outlets are based on services provided [8]. While frequently the franchisor is an NGO, there are a growing number of government and for-profit social franchises.

Social franchising is theorized to increase health care access and utilization by expanding the number of health care outlets and the products and services they offer, and by generating consumer demand through branding and marketing [9], [10]. The dispersed and informal nature of the private sector in developing countries presents challenges in regulating the quality of health services [11]; by organizing independent private providers into a common network franchising facilitates standardization and regulation [9], [12]. Providers are incentivized to join and remain in the network by gaining access to training opportunities, supply of high-quality commodities, and promotional support. These incentives can improve the quality of care and encourage “self-regulation” for complying with quality and affordability standards [13].

### Need for Review

Despite the growth of these programs limited evidence documents their effectiveness. A 2009 systematic review of social franchising did not identify any articles for inclusion [14]. A 2011 scoping review identified twelve articles showing mixed effects of social franchising on patients' perceived quality; and found no association between social franchising and client volume, and limited evidence on the health impact of franchising [15]. A review of reproductive health franchises concluded that franchising is an effective strategy for increasing reproductive health services in the private sector [16]; however an additional review of private sector health interventions reported mixed results on the ability of social franchising to improve quality of care [17]. These reviews report that franchising does not expand access for low-income populations.

Recent advancements in the field of social franchising, paired with an increase in the evaluation of franchising programs, merits an updated review of the literature. In addition, a global consortium of social franchising programs has established a set of programmatic goals for social franchising: quality, health impact, equity, cost-effectiveness, and market expansion [18]. These five goals provide a new framework within which to assess the effectiveness of clinical social franchising.

### Objectives

We assessed the effects of clinical social franchising on clients, communities, and private providers, to better understand the outcomes associated with a strategy currently central to private sector investment in health care in low- and middle-income countries. We evaluated the evidence on each of the goals of social franchising:

- What effect does social franchising have on the quality of health care services?
- What are the health impacts of social franchising?
- What implications do social franchising programs have for the equity of health service delivery?
- Is social franchising a cost effective intervention?
- Does social franchising expand health care access in under-served communities?

## Methods

Peer-reviewed research publications were identified through searching these bibliographic databases: PubMed, Sociological Abstract, EconLit, Social Science Citation Index, Science Citation Index, the World Health Organization's Global Health Library, and the Cochrane Central Register of Controlled Trials. Search terms included a combination of terms on: low- and middle-income countries, social franchising, private sector, and health care. Grey literature was identified through web searches (Google), searching article reference lists, and contacting researchers in the field.

We included all studies of clinical social franchise programs that provided data on at least one of the above outcomes including non-experimental and qualitative studies. Studies of pharmacy franchises or programs that did not provide clinical services were excluded. We included studies set in low- or middle-income countries as defined by the World Bank, and published after 1995. Two independent reviewers scanned the titles of identified records, screened the abstracts and full-text of selected articles, and identified 23 articles for inclusion (NB, DM). Differences in determining eligibility were resolved through review by a third author (AD).

Data was extracted to an Excel database based on guidelines from the Cochrane Handbook for Systematic Reviews of Interventions [19]. Risk of bias in each study was assessed using a 9-point rigour scale for non-randomized studies developed by the WHO-Johns Hopkins Synthesizing Intervention Effectiveness Project. Across studies, there was wide variation in the definitions of study outcomes; thus it was not possible to conduct meta-analysis. A narrative synthesis of data was completed. A research protocol was developed prior to initiation of the review, and followed. The protocol was registered with the PROSPERO database.

## Results

### Study Selection and Characteristics

**Figure 1:**
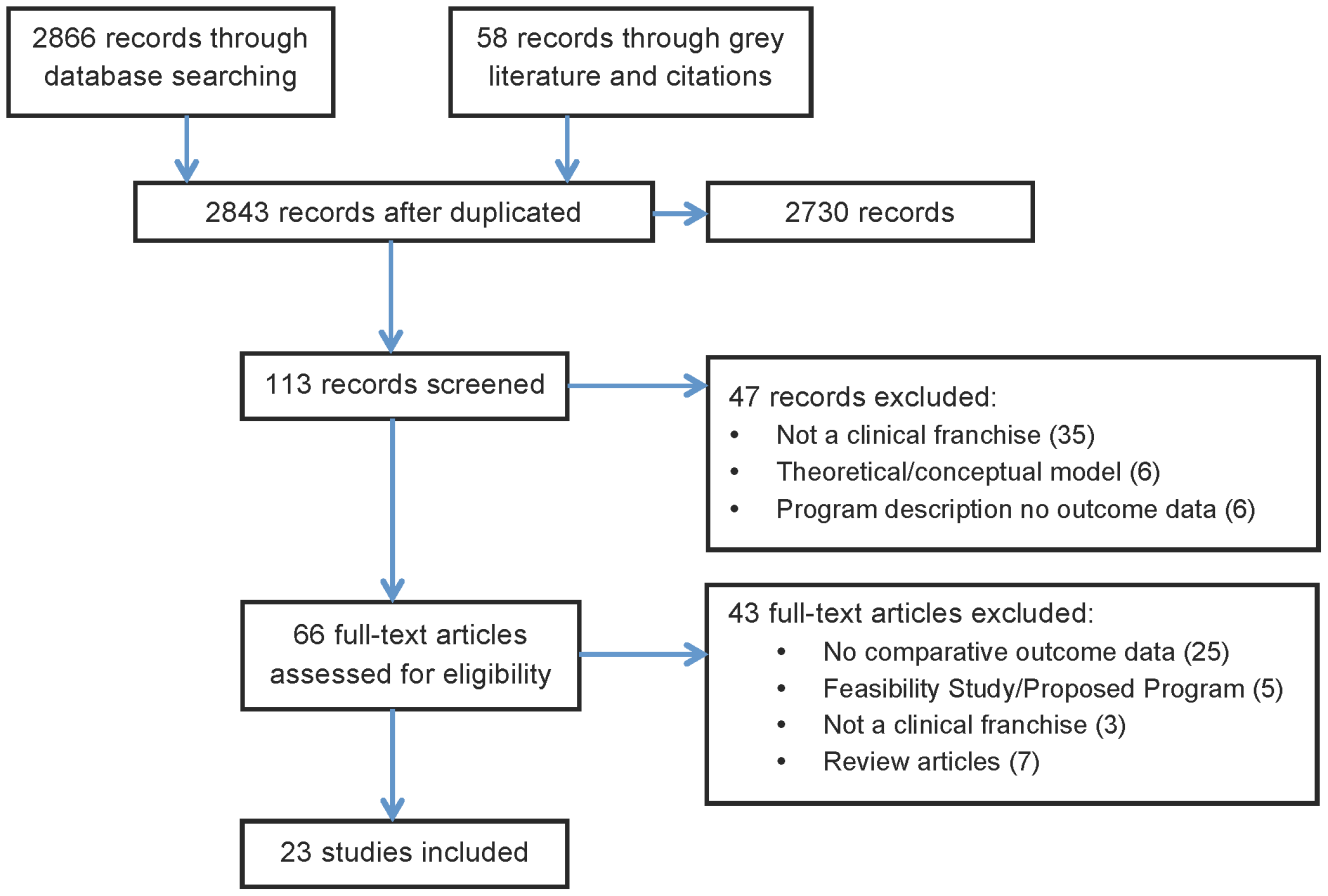
PRISMA Study Selection Flow Diagram.

Of 2,843 records identified, 113 titles were selected for review and 66 read for eligibility. An additional 10 articles were identified through contacting researchers; resulting in 23 selected studies (see Figure 1). These 23 studies included data on nine programs in Africa, South Asia, and Southeast Asia. The majority of these programs (seven) offered reproductive health and family planning services. One program provided child health services, one program provided maternal health services, and one franchise provided reproductive health, child health, and tuberculosis services, and was evaluated on all elements. The studies assessed a number of outcomes that can be grouped according to the five goals of social franchising (see Tables 1, 2, 3, 4, and 5).

**Table 1:** Summary of Health Knowledge and Behavior Findings.

Study	Country	Health Area	Franchise	Study Design	Bias^1^	Main Findings
Agha et al. 2007b	Nepal	Reproductive Health	Fractional	Quasi-Experimental	4	No change in current use of family planning or use of ANC services during last pregnancy in either franchise or control district
Aung et al. in press	Myanmar	Child Health	Full	Cluster Randomized Trial	8	Intervention districts experienced increased use of ORS + Zinc compared to control districts. Intervention districts experienced increased use of ORS, while there was no change in control districts.
Berk and Adhvaryu 2012	Kenya	Child Health		Cross-sectional DHS data analysis	4*	Proximity to franchise clinics increased overall rate of treatment for childhood illness by 14.2%. Slightly increased average number of childhood vaccinations. No association with treatment for diarrhea.
Decker and Montagu 2007	Kenya	Family Planning	Full	Cross-sectional client survey	2	Youth clients at franchise clinics were more likely to use modern family planning methods than youth at non-franchised clinics
Hennink and Clements 2005	Pakistan	Family Planning	Fractional	Quasi-Experimental	6	After introduction of the franchise there was no change in overall contraceptive prevalence rate in intervention communities. There was a shift in contraceptive methods, with increased use of sterilization and decreased condom use. Unmet need for family planning increased in one site and declined in a second site. Compared to control districts, intervention districts experienced increased knowledge of modern family planning methods.
Kozhimannil et al. 2009	Philippines	Maternal Health		Cross-sectional DHS data analysis	4*	Greater exposure to franchise clinics is not associated with any change in the percentage of women receiving an ANC visit in the first trimester or receiving at least 4 ANC visits, but is associated with increased frequency of ANC care. Greater exposure is not associated with any change in the rate of facility delivery, but is associated with increase in delivery in private facility.No change in % of women receiving an ANC visit in the first trimester, or receiving at least 4 ANC visits
Plautz et al. 2003	Madagascar	Reproductive Health	Full	Pre and post household survey with youth	3	Greater exposure to franchise associated with higher self-efficacy for purchase and use of condoms, and higher perceived efficacy of condoms among youth. Greater exposure associated with increased use of condoms among males and increased use of modern contraceptive methods among females.

1 Out of a possible rating of 9, where score of 9 is the least biased. WHO-JHU Synthesizing Intervention Effectiveness Project 9-point rigour scale.

* In the authors' view the use of national survey data to evaluate programs, as applied in these two studies, is inappropriate and highly susceptible to confounding from a number of sources. For these reasons, despite scores of 4 on the rigour scale, we believe the results from these studies should be assessed with caution.

**Table 2:** Summary of Quality Findings.

Study	Country	Health Area	Franchise	Study Design	Bias	Main Findings
Agha et al. 2007a; Agha & Balal 2003	Nepal	Reproductive Health	Fractional	Pre- and post- client exit interviews	3	After introduction of a franchise there is no change in the percentage of returning clients; however there is a significant increase in return visit among educated women. Clients more likely to report attendance at the franchise clinic for reasons related to high quality.
Agha et al. 2007b; Agha et al. 2003	Nepal	Reproductive Health	Fractional	Quasi-experimental	4	Percentage of returning clients increased from 83% to 93% following introduction of the franchise; no change at control clinics. Satisfied clients more likely to return. Client satisfaction increased at intervention clinics from 55% to 77%; no change at control clinics.
Agha et al. 2011	Pakistan	Reproductive Health	Fractional	Cross-sectional provider survey	3	Comparing franchised and non-franchised private providers there was no difference in provider knowledge of IUD insertions or self-efficacy in ability to insert IUD. After controlling for training, there was no difference in number of IUD insertions.
Bishai 2008	Pakistan	Reproductive Health	Fractional	Cross-sectional client and provider survey	2	Franchise clinics are of higher quality than non-franchised private facilities, lower quality than government clinics. Equivalent client satisfaction at franchise and non-franchise clinics.
Decker and Montagu 2007	Kenya	Reproductive Health		Cross-sectional Client Survey	4	Franchise providers more likely to offer targeted family planning for youth than non-franchise providers. Youth at franchise clinics more likely to receive counseling.
Montagu et al. 2005	Nepal	Reproductive Health	Fractional	Cross-sectional Mystery Clients	2	Comparing franchised and non-franchised private providers there was no significant difference in clinic facility quality. Provider practice was poor across all facility types; franchises performed better on some dimensions of care (e.g. privacy), and worse on others (e.g. wait times).
Ngo et al. 2009	Vietnam	Reproductive Health	Gov′ment	Quasi-Experimental	7	After introduction of a new franchise clients have improved perception of staff attitude, no change in perceived quality or staff expertise, and client satisfaction increased. Community has improved perception of overall clinic quality and staff expertise.
O'Connell et al. 2011	Myanmar	Reproductive Health	Fractional	Qualitative	N/A	Clients perceived that SQH clinics are of higher quality, particularly quality of medications, privacy, range of services, technical competency.
Shah et al. 2011	Pakistan Ethiopia	Reproductive Health	Fractional	Cross-sectional client and provider survey	3	In Pakistan franchise clinics are higher quality than non-franchised private clinics and similar quality to public clinics. In Ethiopia franchise clinics are higher quality than non-franchised private clinics and lower quality than public clinics.
Stephenson et al. 2004	Pakistan Ethiopia India	Reproductive Health	Fractional	Cross-sectional client and provider survey	3	Franchises offered more contraceptive brands but had fewer reproductive health services and fewer staff than non-franchise private clinics. Comparing franchised clinics with non-franchised private clinics, client satisfaction was higher in franchised clinics in Pakistan, lower in franchised clinics in Ethiopia, and equivalent across clinic types in India. In Pakistan client willingness to return was higher in franchised clinics than in non-franchised private clinics, while in Ethiopia willingness to return was lower among clients of franchised clinics.

**Table 3:** Summary of Service Utilization Findings.

Study	Country	Health Area	Franchise	Study Design	Bias	Main Findings
Agha et al. 2003	Nepal	Reproductive Health	Fractional	Pre/post- client exit interviews	3	Increase in average daily client volume. No change in percentage of clients using franchised services (reproductive and maternal health)
Huntington et al. 2012	Myanmar	Reproductive & child health	Fractional	Prospective Cohort	2	Average family planning and child health monthly service volume increased, no change in client volume for maternal health services
Lonnroth et al. 2007	Myanmar	Tuberculosis	Fractional	Cross-sectional analysis TB notification data		After launch of TB services, overall notification rate for TB increased. Franchise providers reported 15% of all cases
Ngo et al. 2010	Vietnam	Reproductive Health	Gov′ment	Quasi-Experimental	7	After introduction of a franchise network there was a 40% increase in client volume, 51% increase in client volume for reproductive health, and 45% increase in client volume for family planning In household surveys there was an increase in self-reported frequency of use of franchised services, but no increase in self-reported use.
Qureshi 2010	Pakistan	Reproductive Health	Fractional	Cross-sectional provider survey	2	Franchise affiliation associated with higher weekly client volume
Stephenson et al. 2004	Pakistan Ethiopia India	Reproductive Health		Cross-sectional client and provider survey	3	Franchise associated with higher total client volume and family planning client volume, as compared to non-franchised private clinics.

**Table 4:** Summary of Cost-Effectiveness Findings.

Study	Country	Health Area	Franchise	Study Design	Bias	Main Findings
Bishai et al. 2008	Pakistan	Reproductive Health	Fractional	Cross-sectional client & provider survey	2	Cost per client in franchises lower than government facilities, higher than NGO and non-franchised private. Government facilities include tertiary care centers.
Huntington et al. 2012	Myanmar	Reproductive & Child Health		Prospective Cohort	2	Provider net income increased over the 2-years after joining franchise network
Shah et al. 2011	Ethiopia	Reproductive Health		Cross-sectional client and provider survey	3	In Ethiopia franchise clinics had the highest cost per client. In Pakistan there was no significant difference in cost per client between franchise clinics, government, and non-franchised private clinics. NGOs most cost-effective.

**Table 5:** Summary of Equity Findings.

Study	Country	Health Area	Franchise	Study Design	Bias	Main Findings
Agha et al. 2003	Nepal	Reproductive Health	Fractional	Pre/post client exit interviews	3	After introduction of the franchise the percentage of clients paying 109+ rupees increased from 13–22%. The number of clients reporting that the service costs were ‘moderate’ or ‘high’ increased from 51–96%
Berk and Adhvaryu 2012	Kenya	Child Health		Cross-sectional analysis of DHS data	4	Access, as measured by proximity to franchise, did not vary by household wealth
Bishai et al. 2008	Pakistan	Reproductive Health	Fractional	Cross-sectional client and provider survey	2	Franchise clinics served lower percentage of poor households than non-franchised private providers, higher percentage of poor households than government facilities (gov′t facilities included tertiary care centers)
Hennink and Clements 2005	Pakistan	Reproductive Health	Fractional	Quasi-Experimental	6	Among users of family planning services, women attending franchised clinics were wealthier than women using other sources for family planning.
Montagu et al. under review	Myanmar	Tuberculosis	Fractional	Cross-sectional analysis of TB case records	3	No significant difference between franchise clinics and national sample in percentage of patients in lowest two wealth quintiles. In urban areas, franchise clinics serve a higher proportion of poor clients.
O'Connell et al. 2011	Myanmar	Reproductive health	Fractional	Qualitative focus groups with clients	N/A	Client focus groups report lower fees at franchised clinics than other private clinics
Shah et al. 2011	Pakistan Ethiopia	Reproductive Health	Fractional	Cross-sectional client and provider survey	3	Franchises served fewer low-income people, as compared to public and NGO facilities in Pakistan, and compared to public and non-franchised private clinics in Ethiopia
Stephenson et al. 2004	Pakistan Ethiopia India	Reproductive Health	Fractional	Cross-sectional client and provider survey	3	In Pakistan, higher income people more likely to attend franchised clinics. In Ethiopia and India, no association between client wealth and attendance at franchise clinics.

Few experimental studies of social franchising are available; our review included one cluster randomized trial and one prospective cohort study. Over half of the included studies (thirteen) were cross-sectional surveys of clients and providers or analysis of DHS data; of these studies four analyzed results from a single survey. Five articles presented findings from three quasi-experimental studies. There were two qualitative studies.

### Quality

Over half of the studies measured some aspect of quality making this the most extensively studied outcome of social franchising. However, quality was measured only in family planning clinics, and very few studies assessed quality in a comprehensive manner. Two papers, analyzing data from a multi-country cross-sectional survey, developed a comprehensive quality index using client, provider, and facility surveys and including multiple dimensions of quality. These papers showed that franchised clinics in Pakistan and Ethiopia had higher quality scores than non-franchised private providers but lower quality than government clinics [20], [21]. In Nepal, both franchised and non-franchised clinics had poor facility quality, with no significant difference between clinic types [22].

Three cross-sectional studies assessed provider practice and service availability, dimensions of health care quality known to improve health outcomes and client satisfaction [23]. These studies indicate that franchising has limited effect on clinical quality. A survey in Pakistan found no significant difference in provider knowledge, attitude, or self-efficacy related to IUD insertion between franchised and non-franchised providers [24]. In Ethiopia, India, and Pakistan, franchises had fewer staff and offered fewer reproductive health services, but offered a broader range of contraceptive methods than non-franchised private and government clinics [25]. In Kenya, exit interviews with youth showed that franchised clinics were more likely to offer youth-focused reproductive health services, including family planning counseling; however these findings were not statistically significant [26]. Mystery client visits conducted in Nepal identified no clear difference in quality of care between franchised and non-franchise private clinics; and found provider practice was poor across all clinics evaluated [22].

#### Client Satisfaction and Perceived Quality

The majority of studies on quality measured perceived quality, client satisfaction and willingness to return. Social franchising appears to have a positive effect on all patient-reported quality indicators.

Four studies measured clients' perception of quality. A quasi-experimental study of a government franchise program in Vietnam found that after the introduction of the franchise clients did not report an overall increase in quality, but did perceive improvements in staff attitude. At the community level, residents perceived improvements in both overall service quality and provider expertise [27]. Qualitative focus groups reported similar findings; clients in Myanmar perceived franchise clinics to be of higher quality, in particular offering safer drugs and more privacy [28] and in Vietnam reported improvements in the quality of staff and facilities after introduction of franchised services [29]. However cross-sectional survey data showed no significant difference in perceived quality between clients of franchised and non-franchised clinics in India, Pakistan, and Ethiopia [25].

Social franchising may have a positive effect on client satisfaction with clinic services. Quasi-experimental pre- and post-studies show increased client satisfaction at a government franchise in Vietnam [27], and a non-profit franchise in Nepal [30]. Analysis of cross-sectional exit interview data found no variation in overall levels of client satisfaction between franchised and non-franchised clinics in India and Pakistan [20], [25]; however in Pakistan, franchise clients were more satisfied with the range of contraceptive methods and services offered [20]. Only one study documented lower client satisfaction among franchise clients [25].

There is less clear evidence on the effect of social franchises on clients' willingness to return. The most rigorous study of client loyalty found significant increases both in client willingness to return and willingness to recommend the franchise clinic to others following the introduction of a government franchise [27]. Other studies showed divergent results. In Nepal, one study found an increase in the percentage of returning clients [30], while a second study of the same franchise showed no change [31]. Likewise, Stephenson et al found that franchise clients in Pakistan were more likely to report willingness to return while in Ethiopia franchise clients were significantly less willing to return, as compared to clients at other private or public facilities [25].

### Health Impact

Research on the health impacts of social franchising at both the client and community levels focus on service utilization and health behaviors. No studies evaluate the health outcomes associated with social franchise programs.

#### Health Care Service Utilization

Social franchising increases client volume and service utilization. A prospective cohort study in Myanmar showed increasing monthly client volume for family planning services for the first 4-years after joining a franchise network. Client volume also increased for child health services [32]. Another prospective study in Vietnam found franchise membership increased total client volume by 40%, and use of reproductive health services by 51% [33]. In India, Ethiopia, and Pakistan franchise clinics had higher client volume and family planning client volume than non-franchised private providers [25].

Seven studies evaluated the community-level effects of social franchising on utilization of health services with positive effects observed in child health and tuberculosis services. The strongest evidence comes from a cluster randomized trial in Myanmar where the introduction of a new franchise network increased the use of ORS + Zinc in the treatment of childhood diarrhea, and increased the number of caregivers seeking medical care for their children [34]. Analysis of DHS data in Kenya showed that children living near a franchise clinic were more likely to receive treatment for malaria, and received slightly more vaccinations, than children living a greater distance from franchised clinics [35]. The addition of tuberculosis services to a franchise in Myanmar improved TB reporting; the franchise contributed 15% of all TB case notification [36].

However social franchising has not been shown to increase utilization of maternal or reproductive health services. In Vietnam, household surveys showed that increases in client volume did not correspond to expanded health access at the community level. There was no increase in the rates of self-reported clinic use but a significant increase in self-reported frequency of use, indicating that client volume increases were the result of increasing visits per client [33]. Studies in Nepal showed different results on client volume for franchised services; one study documented an increase in the percentage of clients using franchised reproductive health services from 19 to 26% [31] while a second study showed that clients using these services remained unchanged even as total client volume increased [37]. Analysis of DHS data in the Philippines showed no association between the presence of franchise clinics and increased use of antenatal care services or facility deliveries [38].

#### Health Behaviors

Studies measuring health knowledge and behavior show positive effects among franchise clients, but no significant impacts at the community level. In Kenya youth attending franchise clinics were more likely to use modern methods of family planning then youth attending non-franchised clinics [26]. In Madagascar, youth with greater exposure to a social franchising and social marketing intervention had higher knowledge about family planning and STI prevention, and increased self-efficacy for the purchase and use of condoms. Exposure to the program also increased use of modern family planning; however the intervention did not increase the utilization of health services for sexually transmitted infections [39]. These individual-level changes do not necessarily result in significant population-level impacts. Household surveys conducted before and after the introduction of reproductive health franchises in Nepal and Pakistan found no change in contraceptive prevalence rate [30], [40].

### Equity

Equity measures included two dimensions: the comparative wealth distribution of clients at franchised and non-franchised clinics, and the cost and perceived affordability of franchised services. Social franchises serve relatively higher income clients and franchising results in higher service costs to consumers. Household surveys near franchises in Pakistan found that wealthier women were more likely to attend franchise clinics while poorer women were more likely to seek services at non-franchised clinics [40]. Exit interview data from Pakistan showed similar results [20], [21], [25]. In Ethiopia, franchised clinics also served higher income clients than non-franchised private providers [21]. A single study in Myanmar, comparing clients of franchised TB services to a nationally representative sample of TB patients, found that franchise clinics served a higher proportion of low-income clients in urban areas; however, in rural areas and at a national level, there was no significant difference in the client wealth profile of franchised and national samples [41].

A single study evaluating service costs in Nepal showed that introducing franchised services increased service charges and increased the number of clients perceiving charges as moderate or high [37].

### Cost-Effectiveness

A multi-country cross-sectional survey provides the only available data on the relative cost-effectiveness of providing franchised services. This study calculated cost efficiency as the cost per client of providing services including salary and rent, and excluding commodity costs. In Ethiopia, franchises had the highest cost per client for providing care, as compared to non-franchised providers, NGO and government clinics [21]. In Pakistan, there was no significant difference between the cost per client at franchised clinics, non-franchised private clinics and government clinics [20], [21].

### Market Expansion

No studies of social franchising have investigated the impact of this intervention on the total availability of health services.

### Risk of Bias

These findings are based on largely low-quality data. To assess the risk of bias in each study we used the WHO-Johns Hopkins University Synthesizing Intervention Effectiveness Project 9-point rigour scale [42]. The scale evaluates studies on a number of dimensions; the inclusion of pre- and post-intervention data, presence of a control group or cohort, equivalency of comparison groups, random assignment to the intervention and selection for assessment, adequate consideration of confounding factors, and follow-up. Sixteen of the studies had a score of four or lower; the largely cross-sectional data did not include pre- and post-assessment data and few studies randomized participation in the interventions or discussed equivalency between comparison groups. Only three studies received a score of six or higher. The low quality of evidence, resulting from issues such as poor study design and heavy reliance on patient self-reported data, significantly limits the ability to draw strong conclusions from this data. The wide variation in study design and in the definition and measurement of outcomes prevented assessing the strength of evidence by outcome.

## Discussion

Given the large role of the private sector health care delivery in developing countries and the substantial concerns about the quality and accessibility of these services, social franchising is widely believed to be a promising intervention to strengthen private sector health services. International donors, governments, and franchise program implementers have turned growing attention toward evaluating the effectiveness of clinical social franchises in meeting health care needs in developing countries. A global consortium of social franchise programs established common goals for social franchises [18], providing a framework within which to evaluate the future development of franchise programs.

We find limited and mixed evidence on the achievement of these goals as a result of the minimal and low quality research. Available research emphasizes elements of quality and health impact, demonstrating that social franchising increases client volume and client satisfaction, and in some settings improves client health knowledge and behavior. Yet there is little research that documents a positive effect of franchising on improving health care quality or equity, or achieving improvements in population-level health outcomes.

Our review highlights several remaining gaps in knowledge about the effect of social franchising, which should be addressed to inform program and policy development. First, although quality was the most extensively studied outcome, the majority of the research focused exclusively on client satisfaction without addressing dimensions of quality that are known to improve health outcomes, such as provider technical competence, and the quality and availability of essential equipment and medications [43]–[45]. Although franchising is theorized to improve quality through improved monitoring and oversight, this can be challenging in large franchise networks [10], [46], and there is limited evidence that franchising improves clinical quality. As researchers develop tools for measuring quality in low-resource settings [47], attention should focus on the quality impacts of social franchising, as well as the organizational and policy environments that facilitate franchises reaching their quality goals.

Second, research should consider the health impacts of social franchising. Social franchising increased utilization of child health services in Myanmar [34] and Kenya [35], however studies of reproductive and maternal health franchises did not find any increase in health service use. This is surprising given that the majority of social franchise networks provide primarily reproductive health services. Future research should include expanded investigation of the impacts of social franchising on health behaviors and health outcomes.

There is no evidence on the ability of clinical social franchising to expand the availability of health services in currently underserved areas. Social franchising can, in theory, increase the number of providers particularly where the existing medical workforce is under-utilized [16], [48]. However, some evidence suggests that social franchises do not substantially expand access to health services but rather recruit existing providers into the network or shift users from one source of care to another [49]. Future research should investigate the substitutive impacts of social franchising to understand the programs' impact on expanding access to healthcare services. Greater attention should also be paid to the effects of social franchising on the health system, for instance evaluating effects on non-franchised private and public clinics.

Finally, continued research is needed to understand the equity impacts of franchising. Social franchise implementers have a clear goal of serving low-income populations; however franchised clinics serve a greater proportion of higher income clients than other facility types. It is assumed that the location of franchised clinics in low-income communities is synonymous with serving the poorest households yet this review finds that geographic location in a poor area does not result in equitable access by wealth. The introduction of franchised services can increase costs and reduce perceived affordability of health care [30], in line with prior reviews documenting higher costs in the private sector [3], [50]. In fractional franchise networks the quality and price controls only affect the franchised services while providers can use the franchise brand name to draw customers to the full range of services offered. In Myanmar providers who joined a franchise network increased their income; largely by increasing the number of clients accessing non-franchised, and therefore non-price controlled, services [32]. Future research into the effects of franchising on the price of health services, as well as how this shapes who accesses social franchise services may enable programs to achieve greater progress towards their equity goal.

This review suggests some contexts within which franchising is an effective intervention, and can guide future program development. While franchises are often of equivalent or lower quality than public clinics [20], [25], they are typically of higher quality than non-franchised private providers [21], [25]. Franchising may be a particularly useful strategy in areas where a large unregulated private sector provides the majority of health services. Franchising can also be implemented effectively by governments to strengthen public sector health care delivery [27], [33], and is an efficient way to introduce new services into existing private practices. Our review also highlights new health service areas that are successfully being delivered in the franchising model, such as child health and TB services, suggesting the need for continued expansion of social franchising beyond the traditional emphasis on reproductive health care. Understanding where social franchises can play the greatest role in improving health care delivery, and which models of franchising are the most effective in improving health outcomes, will more effectively target services.

### Limitations

This review had several limitations. We chose to include non-experimental and qualitative studies, and the low quality of many of the studies limits our ability to draw strong conclusions. A number of the included studies analyzed data from a single survey further limiting the scope of evidence [20], [21], [25], [51]. The studies evaluate a small set of social franchising programs globally and the results are heavily influenced by findings from just a handful of programs: the majority of negative results are from a program in Ethiopia, while the strongest studies and most positive results are from a franchise in Myanmar. The studies in this review also focus almost exclusively on reproductive health franchises, and do not represent the range of franchise programs currently operating globally, making these results difficult to generalize to the full range of social franchising programs. Finally, we included only English-language articles in this review.

### Conclusion

In recent years the private sector has grown to become a major source of health care in low- and middle-income countries, as a result of many factors including declining government funding and patient preference for private-sector services [3]. Working with the private sector is essential to improve the quality and delivery of health care services; however, our findings on quality, equity and community-level health impacts indicate the need for a continued focus on program development in order for social franchising to contribute significantly to strengthening the private sector. The creation of international goals and standardized metrics for clinical social franchising demonstrates the interest of donors and program implementers in understanding program effectiveness. As research and evaluation continue to document the effects of social franchising and the contexts within which this model is successful, it remains to be seen how social franchising programs will respond to the existing limitations, and the role franchising may continue to play within the health systems in low- and middle-income countries.
